# Ontogenetic shifts in olfactory rosette morphology of the sockeye salmon, *Oncorhynchus nerka*

**DOI:** 10.1002/jmor.21539

**Published:** 2022-12-11

**Authors:** Sarah E. Rheinsmith, Thomas P. Quinn, Andrew H. Dittman, Kara E. Yopak

**Affiliations:** ^1^ Department of Biology and Marine Biology University of North Carolina Wilmington Wilmington North Carolina USA; ^2^ School of Aquatic and Fishery Sciences University of Washington Seattle Washington USA; ^3^ Environmental and Fisheries Sciences Division, Northwest Fisheries Science Center, National Marine Fisheries Service, National Oceanic and Atmospheric Administration Seattle Washington USA

**Keywords:** neurobiology, olfactory rosette, ontogentic shifts, sockeye Salmon

## Abstract

Sockeye salmon, *Oncorhynchus nerka*, are anadromous, semelparous fish that breed in freshwater—typically in streams, and juveniles in most populations feed in lakes for 1 or 2 years, then migrate to sea to feed for 2 or 3 additional years, before returning to their natal sites to spawn and die. This species undergoes important changes in behavior, habitat, and morphology through these multiple life history stages. However, the sensory systems that mediate these migratory patterns are not fully understood, and few studies have explored changes in sensory function and specialization throughout ontogeny. This study investigates changes in the olfactory rosette of sockeye salmon across four different life stages (fry, parr, smolt, and adult). Development of the olfactory rosette was assessed by comparing total rosette size (RS), lamellae number, and lamellae complexity from scanning electron microscopy images across life stages, as a proxy for olfactory capacity. Olfactory RS increased linearly with lamellae number and body size (*p* < .001). The complexity of the rosette, including the distribution of sensory and nonsensory epithelia and the appearance of secondary lamellar folding, varied between fry and adult life stages. These differences in epithelial structure may indicate variation in odor‐processing capacity between juveniles imprinting on their natal stream and adults using those odor memories in the final stages of homing to natal breeding sites. These findings improve our understanding of the development of the olfactory system throughout life in this species, highlighting that ontogenetic shifts in behavior and habitat may coincide with shifts in nervous system development.

## Graphical abstract

Scanning electron microscope images of sockeye salmon olfactory rosette. Olfactory rosette size linearly increases with lamellae number throughout ontogeny. The complexity of the rosette, including the distribution of sensory and nonsensory epithelia, and the appearance of secondary lamellar folding, varies between fry and adult life stages. These differences in epithelial structure may indicate variation in odor‐processing capacity between juveniles and adults.

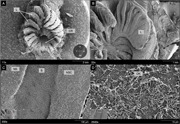

## 1 INTRODUCTION

Olfaction is a critical sense in the aquatic environment, as many species rely on chemosensory cues for navigation and homing (Hasler & Scholz, 1983; Nosal et al., 2016; Werner & Lannoo, 1994), identification of odors related to conspecifics and reproduction (Olsén & Liley, 1993; Stacey, 2003), kin recognition (Brown & Brown, 1996), feeding (Hara, 1982; Kasumyan, 2004), predator avoidance (Chivers & Smith, 1993; Døving & Lastein, 2009), and larval settlement (Bilodeau & Hay, 2021). Given the critical role of olfaction in all aspects of their life history, morphological variations in the olfactory system have been investigated in detail in fishes.

The olfactory system of salmonids has been given particular attention, given their unusual life history: embryos hatch in freshwater, emerge as fry from their gravel nests, and then rear in freshwater (parr stage) until undergoing a series of changes in physiology, morphology, and energetics, termed smolt transformation, that prepare them to migrate downstream from the lake and make the transition from fresh to salt water (Hoar, 1976). During their adult life stages, they migrate long distances back to their natal stream, relying on odor memories to return to the natal site for spawning, years after they left it as juveniles. Thus, the olfactory system must be sufficiently developed at early juvenile stages to acquire chemosensory information needed for learning (imprinting to) natal stream odors that are used later to guide homing migrations (Barnett et al., 2019; Brannon, 1972; Quinn et al., 2006; Zielinski & Hara, 1988).

For *Oncorhynchus* spp., it is proposed that individuals imprint during early development on odors, including dissolved free amino acids, specific to their natal streams (Shoji et al., 2000, 2003; Ueda, 2012), which leads to a memory‐associated migration (Ueda, 2019). This imprinting period likely occurs during embryonic development (Dittman et al., 2015; Tilson et al., 1994), before the parr‐smolt transformation (Shrimpton et al., 2014), and again during the smolt life stage (Havey et al., 2017). Later, during the homeward migratory phase, maturing adults use these imprinted odors, perhaps augmented with odors from conspecifics (Bett & Hinch, 2016), to discriminate their natal stream from others with high precision (Dittman & Quinn, 1996; Hasler & Scholz, 1983).

**Figure 1:**
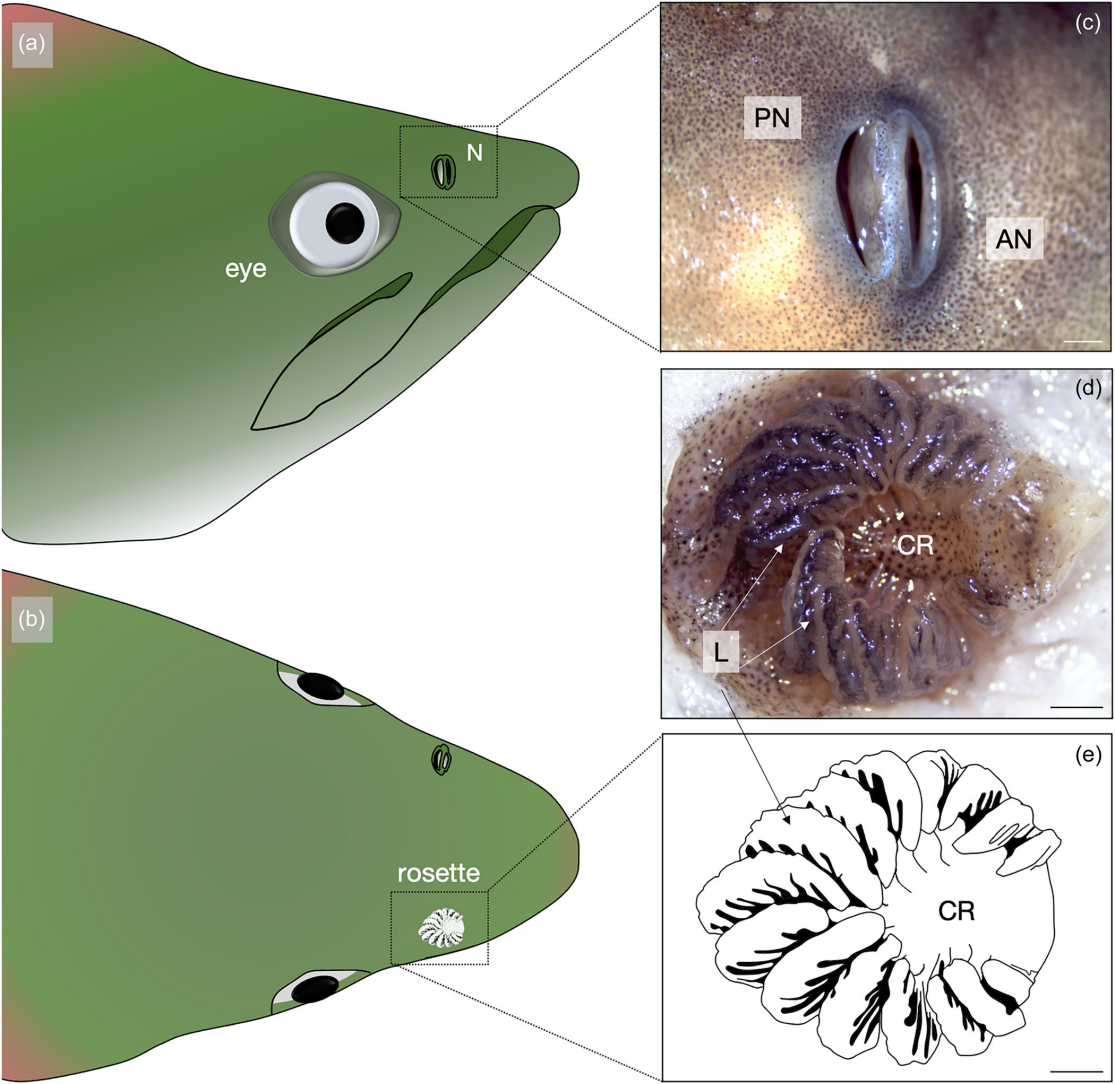
*Oncorhynchus nerka*, diagram of the anterior head of a representative migrating adult, male sockeye. (a) In lateral and (b) dorsal views and the anatomical structure of the (c) external and (d and e) internal olfactory organ, including (e) a graphical representation of the adult olfactory rosette, showing the prominent secondary folding. AN, anterior nare; CR, central raphe; L, lamellae; N, nare; PN, posterior nare. Scale bar = 1 mm.

In salmonids, the detection of chemosensory cues is governed by a well‐developed peripheral sensory organ, the olfactory rosette, and a relatively large olfactory bulb (the first‐order olfactory processing center in the brain; Døving et al., 1985; Wisby & Hasler, 1954). The olfactory organs in teleost fishes are found in paired nasal cavities (nares), situated on the dorso‐rostral end of the snout (Hara, 1994; Kermen et al., 2013) (Figure 1). There are two olfactory rosettes (left and right), each positioned internally between the anterior and posterior nares. Each olfactory rosette is comprised of a central raphe that is surrounded by olfactory lamellae on either side (Figure 1; Hansen & Zeiske, 1998, Hansen & Zielinski, 2005; Zeiske et al., 1992). An individual lamella is made up of a sensory epithelium that develops to create two layers of olfactory lamellae: primary and secondary (Calvo‐Ochoa & Byrd‐Jacobs, 2019; Fishelson et al., 2010; Zeiske et al., 1992). In teleosts, primary lamellae comprise the olfactory epithelium, which contain the olfactory receptor neurons (ORNs), responsible for the recognition of biologically relevant, water‐borne odorants (Zeiske et al., 1992). Secondary lamellae, present in some groups of teleosts (Kasumyan, 2004), are described as the lamellar folds present on the primary lamellae.

There is high inter‐ and intraspecific variability in the size and complexity of the nervous system in all major fish groups, often correlated with their ecology and life history (e.g., Angulo & Langeani, 2017; Bauchot et al., 1979; Laforest et al., 2020; Pollen et al., 2007; Salas et al., 2017; Wagner, 2003; Yopak, 2012). Correspondingly, there is considerable variation in the morphology of the olfactory organs within and across fish species, including rosette size (RS) and shape, number, shape, and arrangement of lamellae, and ORN number (Abe et al., 2020; Dymek et al., 2021; Ferrando et al., 2019; Fishelson et al., 2010; Ghosh, 2020; Hara, 2011; Kasumyan, 2004; Kudo et al., 2009; Zeiske et al., 1992).

In salmonids, the importance of odors for homing suggests that changes in the size and complexity of the olfactory system at key life stages may parallel variation in olfactory processing and/or capacity. Early olfactory rosette development may occur before hatching (Brannon, 1972; Zielinski & Hara, 1988), but continues throughout early life stages, with variation in rosette morphology across ontogeny. Lamellae count increases markedly across ontogeny in chum salmon (*O. keta)* (Kudo et al., 2009) and the absolute number of ORNs in the sensory epithelium changes from hundreds of thousands in fry to tens of millions of cells in mature adults of this species (Kalinina et al., 2005; Kudo et al., 2009; Yamamoto & Ueda, 1977). Surprisingly, there have been no studies to quantify how olfactory function covaries with anatomical differences in salmon. However, morphological changes in the olfactory system may reflect differences in olfactory capacity (as part of the imprinting or odor‐recall process) in salmon, corresponding to discrete life stages and varying habitats across ontogeny.

This study assessed morphological differences in the olfactory rosette of sockeye salmon across a series of life stages: fry that had just entered the lake, parr feeding in the lake, smolts leaving the lake a year later, adults that have left the ocean and are migrating into their natal river system, maturing adults, and fully mature fish entering the spawning stream itself. The gross morphology and ultrastructure of the olfactory rosette and the sensory portions of the olfactory epithelium was assessed using scanning electron microscopy (SEM). This study tested the hypotheses (1) that the relative size of the olfactory rosette increases throughout ontogeny, (2) that there is an increase in primary lamellae number across the life stages, and (3) that there is an increase in lamellar complexity, as defined by anatomical changes to the sensory epithelium and the presence of secondary folding.

## 2 MATERIALS AND METHODS

### 2.1 Specimen collection

A total of 26 specimens of *Oncorhynchus nerka* (Walbaum, 1792) across different life stages were collected from Lake Aleknagik, Alaska, and Hansen Creek, one of its tributaries, according to the ethical guidelines of the University of Washington (IACUC protocol #3142‐01). Specimens were categorized based on their developmental stage (Groot & Margolis, 1991) and included fry (*n* = 3), parr (*n* = 4), smolt (*n* = 5), and adults (*n* = 12). Samples (fry‐maturing adults) were collected from Lake Aleknagik, whereas spawning adults were collected at Hansen Creek, a tributary of the lake, in late July (Table 1). Fry, parr, and smolt specimens were collected using a seine net, tow net, and fyke net, respectively. Adults (six males and six females) represented three points along their migratory continuum: “migrating” (just entered freshwater) and “maturing” (transitioning in freshwater to maturity), both collected with gill nets, and “spawning” adult (fully mature, ready to spawn, and entering their natal stream), collected in Hansen Creek itself. Small fishes (fry, parr, smolt) were euthanized via an overdose of MS‐222 (m‐aminobenzoic acid ethyl ester, methansulfate salt), buffered to neutral pH, and adults were euthanized with a sharp blow to the head. Upon collection, morphometric measurements for each individual were recorded, including body mass (g; Table 1). Immediately after euthanasia, individuals were immersion‐fixed in 10% neutral buffered formalin and postfixed for up to 36 months.

**Table 1:** The collection date (all in 2016), sample size, average body mass (g ± SD), average lamellae count (±SD), and rosette size (mm^2^ ± SD) for fry, parr, smolt, and adult life history stages

Life history stage	Date	Sample	Body mass (g)	Lamellae count	Rosette size
Fry	June 16	3	1.1 ± 0.69	5.0 ± 1.0	0.32 ± 0.15
Parr	August 28	4	2.8 ± 0.78	7.0 ± 0.8	0.95 ± 0.41
Smolt	June 12	5	7.2 ± 0.96	10.4 ± 1.5	1.44 ± 0.15
Migrating adult	June 21	5	1600 ± 220	14.6 ± 1.1	27.2 ± 4.06
Maturing adult	July 10	2	1750 ± 212	14.0 ± 1.4	24.8 ± 1.53
Spawning adult	July 24–29	5	1570 ± 103	14.6 ± 0.6	26.3 ± 2.96

*Note*: Adults are separated in sample collection based on when they entered their natal watershed. Migrating adults had recently entered freshwater. Maturing adults had spent ~1 month in their natal lake continuing to mature and spawning adults were recovered in their natal stream at final maturation. For statistical analyses, all adults were pooled together.

### 2.2 Olfactory rosette preparation

Based on previous research suggesting no bilateral differences between the left and right olfactory structures in fishes (e.g., Camilieri‐Asch, Shaw, et al., 2020), the left rosette was arbitrarily dissected from each specimen to assess its morphology. Following postfixation, the olfactory rosette was separated from the olfactory nerve (nI) and immersed in a 0.1 mol L^−1^ phosphate buffer solution (PBS) for 24 h. Rosettes were then submerged in 1:1 ratio of osmium tetroxide in a 0.1 mol L^−1^ PBS for 1 h. Samples were then washed with deionized (DI) water to remove excess fixative and dehydrated through a classic graded ethanol series (20 min for each series). After dehydration, samples were placed in 100% ethanol, critical point dried, and sputter coated with 13.1 nm platinum.

### 2.3 SEM and morphological assessment

Variations in olfactory rosette morphometrics throughout ontogeny were assessed using a ApreoS HiVac SEM, at a working distance of approximately 10 nm, a beam strength of 5 kEv, and a 13‐spot size. Low (×10–×100) and high (×500–×10,000) magnification images were acquired to estimate RS and assess its organization and composition (as proxies for complexity) across individuals and life stages. Data on RS and lamellar number were acquired from low magnification images for fry, parr, smolt, and adults. However, comparisons of rosette complexity from high magnification images are restricted to fry and adult life stages only, as higher magnification imaging of the sensory epithelium for parr and smolt was not possible, due to damage to the epithelia during tissue processing. Assessment of lamellae at low and high magnification was conducted on an intact rosette. The digital images acquired were saved/downloaded as (1536 × 1094, RBG Gray, uncompressed), exported as.TIFF files, and assessed in Adobe Photoshop®.

For all samples, the gross morphology of the rosette was assessed and lamellae number and total olfactory RS (in mm^2^) were measured. For all samples, rosette ultrastructure was assessed from digital images in Adobe Photoshop® to count the number of lamellae in each specimen and calculate size of the total olfactory rosette (in mm^2^). As the olfactory organ is cylindrical and dorsoventrally flattened in shape, total RS was calculated from an image of the dorsal face of the rosette (i.e., the portion exposed to the incurrent opening of the nare), captured with SEM. RS was calculated by approximating the shape of the rosette as an oval. A digital overlay of the oval shape was delineated in Adobe Photoshop® and included all epithelial tissue across the multiple lamellae, but excluded bony structures and epineurium in the olfactory cavity. Using the Ruler Tool, a measurement scale was set by assigning a specified number of pixels per number of scale units (mm) for each image. RS was then extrapolated by counting the total number of pixels within the overlain oval, converted to area (mm^2^).

### 2.4 Statistical analysis

To test for differences in the olfactory rosette across different life stages, the number of lamellae and total olfactory RS were averaged, and standard deviations (±SD) reported for each life stage (fry, parr, smolt, and adult). These parameters were compared between life stage categories using an analysis of variance. For purposes of statistical analyses, all three adult stages were pooled because they did not differ in RS (*F*(1, 10) = 0.185, *p* = .676) and lamellae count (*F*(2, 9) = 0.321, *p* = .733) across adult life stages (Table 1).

To assess the scaling relationship between RS and body size throughout ontogeny, RS was scaled against body mass using an ordinary least squares (OLS) linear regression (*y* = *ax* ^*b*^). Before analysis, body mass (*x*) and RS (*y*) were log_10_‐transformed. A qualitative description of the sensory epithelium was then performed on SEM images, to identify the boundary between sensory versus nonsensory epithelia (×10–×1000 magnification), the presence of ORNs (apical olfactory knobs [OKs]; ×2500), and, when possible, morphological differences between the OKs at the epithelial surface (at ≥×2500) of fry and adult individuals. Note that individual lamellae were not able to be nondestructively removed from the central raphe and assessment of the lamellae (quantitative and qualitative) were performed while positioned on the intact rosette. All quantitative statistical analyses were conducted in R (R Core Team, 2020).

## 3 RESULTS

### 3.1 Gross morphology of the olfactory rosette

**Figure 2:**
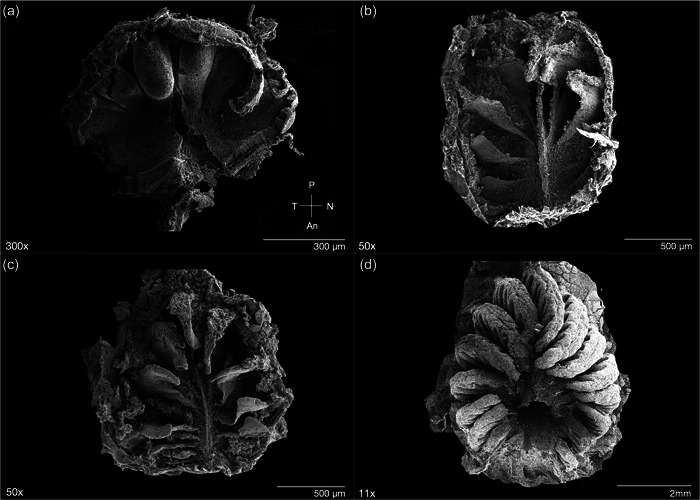
*Oncorhynchus nerka*, scanning electron micrographs of the olfactory rosette of representative (a) fry, (b) parr, (c) smolt, and (d) adult specimens. Rosettes are positioned internally between the anterior and posterior nare. Each rosette possesses a central raphe with protruding arrow‐like lamellae, initiating just anterior to from the center of the rosette. Orientations shown in (a) indicate anterior (An), nasal (N), posterior (P), and temporal (T) position of the left rosette within the nasal cavity across all panels.

Sockeye salmon (*O. nerka*) have paired olfactory organs located inside the olfactory cavity on either side of the head. The arrangement and shape of the lamellae forming the rosette was consistent across all life stages and thus did not change throughout ontogeny. This species exhibits an “arrow‐like” olfactory organ, similar to other salmonids (see review by Kasumyan, 2004). Specifically, in an oval‐shaped rosette, lamellae are positioned radially around a central raphe, and the “origin” of the radiation was slightly anterior to the oval center; further, lamellae appeared to increase in size incrementally from the anterior to posterior ends of the rosette (Figures 1 and 2). For each lamella, it is larger at the origin from the median raphe and tapers toward the edges of the rosette, at the outer lamellar margin (Figure 2). There was a visible increase in lamellar complexity and RS at the adult stage compared to earlier life stages. Adult specimens displayed prominent secondary folding (secondary lamellae) that have both sensory and nonsensory epithelial regions (Figures 2d and 5c); but, as individual lamellae could not be isolated, percent cover could not be quantified. In contrast, juveniles (fry, parr, and smolt) showed no visible secondary lamellae (Figure 2a–c). In fry, distinct regions of sensory versus nonsensory epithelia could not be visualized on the lamellar surface at high magnification. Rather, only sensory epithelia were observed across the exposed portions of the lamellae (Figure 2a).

### 3.2 Lamellae count and RS

**Figure 3:**
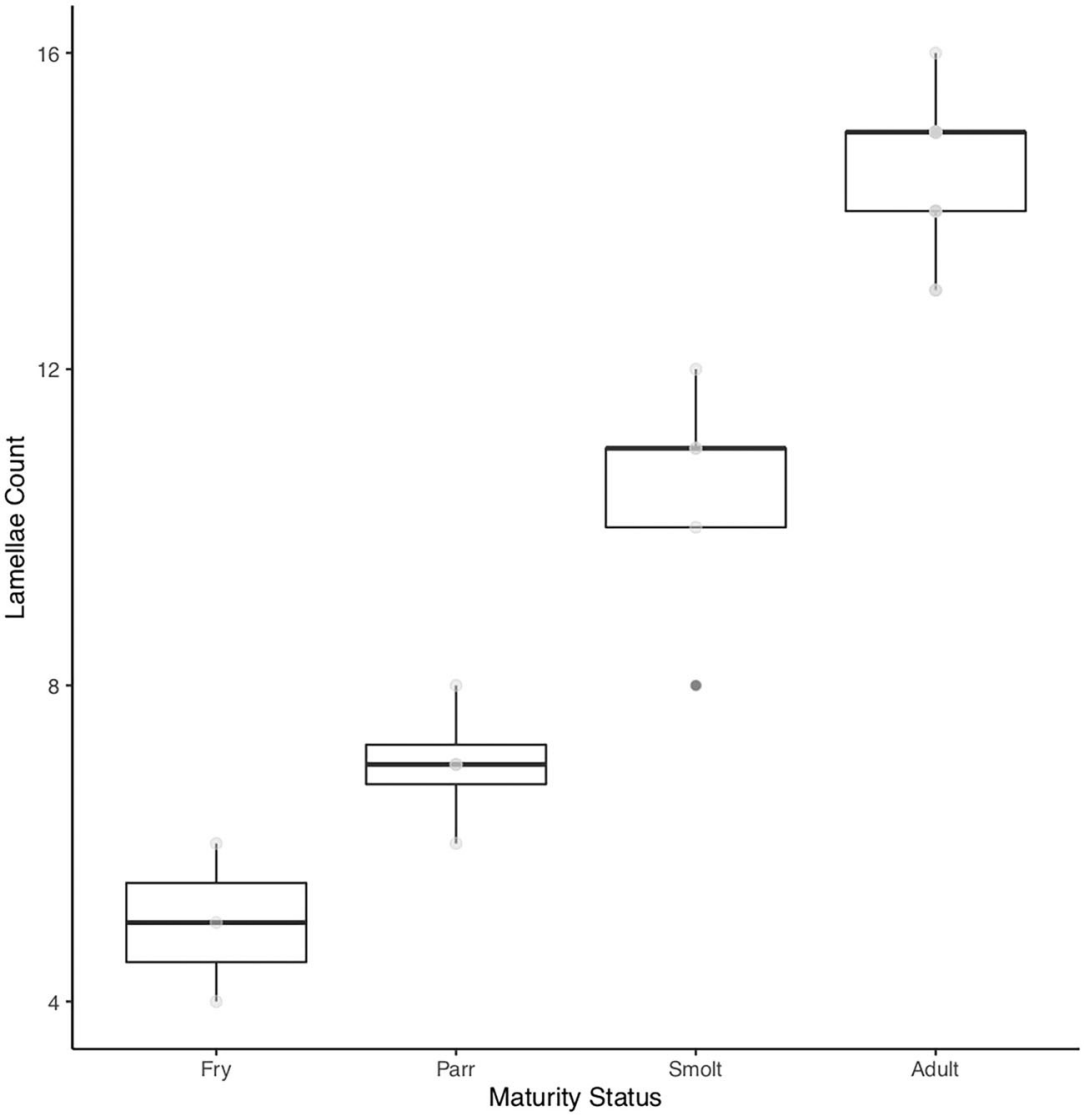
Box and whisker plot showing changes in lamellae number between fry, parr, smolt, and adult sockeye salmon. There were significant changes in lamellae number between life stages (*F*(3, 20) = 96.02, *p* = 4.773e^−12^).

**Figure 4:**
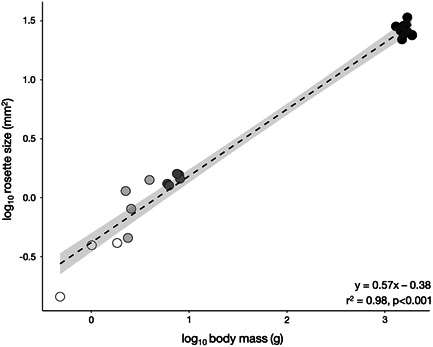
Scaling relationship between and log_10_‐transformed rosette size (mm^2^) and log_10_‐transformed body mass (g) of sockeye salmon across four different life stages indicated by color: white = fry, light gray = parr, dark gray = smolt, and black = adult. Gray area represents 95% CI (0.530, 0.600).

The average number of lamellae increased throughout ontogeny, from fry to adult stages (Table 1; Figure 3; L_FRY_ = 5.00 ± 0.31, L_PARR_ = 7.00 ± 0.95, L_SMOLT_ = 10.40 ± 1.52, L_ADULT_ = 14.50 ± 0.90) and differed significantly between life stages (Figure 3; *F*(3, 20) = 96.02, *p* = 4.773e^−12^). The average total size of the olfactory rosette (Table 1; RS_FRY_ = 0.32 mm^2^ ± 0.15, RS_PARR_ = 0.95 mm^2^ ± 0.41, RS_SMOLT_ = 1.44 mm^2^ ± 0.15, RS_ADULT_ = 26.47 mm^2^ ± 3.18) also increased significantly from earlier to later life stages (*F*(1, 22) = 1116, *p* = 2.2e^−16^). In addition, the total RS increased significantly with body mass throughout ontogeny (Figure 4; *y* = 0.57*x* − 0.38, *r* ^2^ = .98, *p* < .001) and scaled with a negative allometry; the rosette increased in size throughout life, but grew at a slower rate than the rest of the body.

### 3.3 Sensory epithelia in fry and adult stages

**Figure 5:**
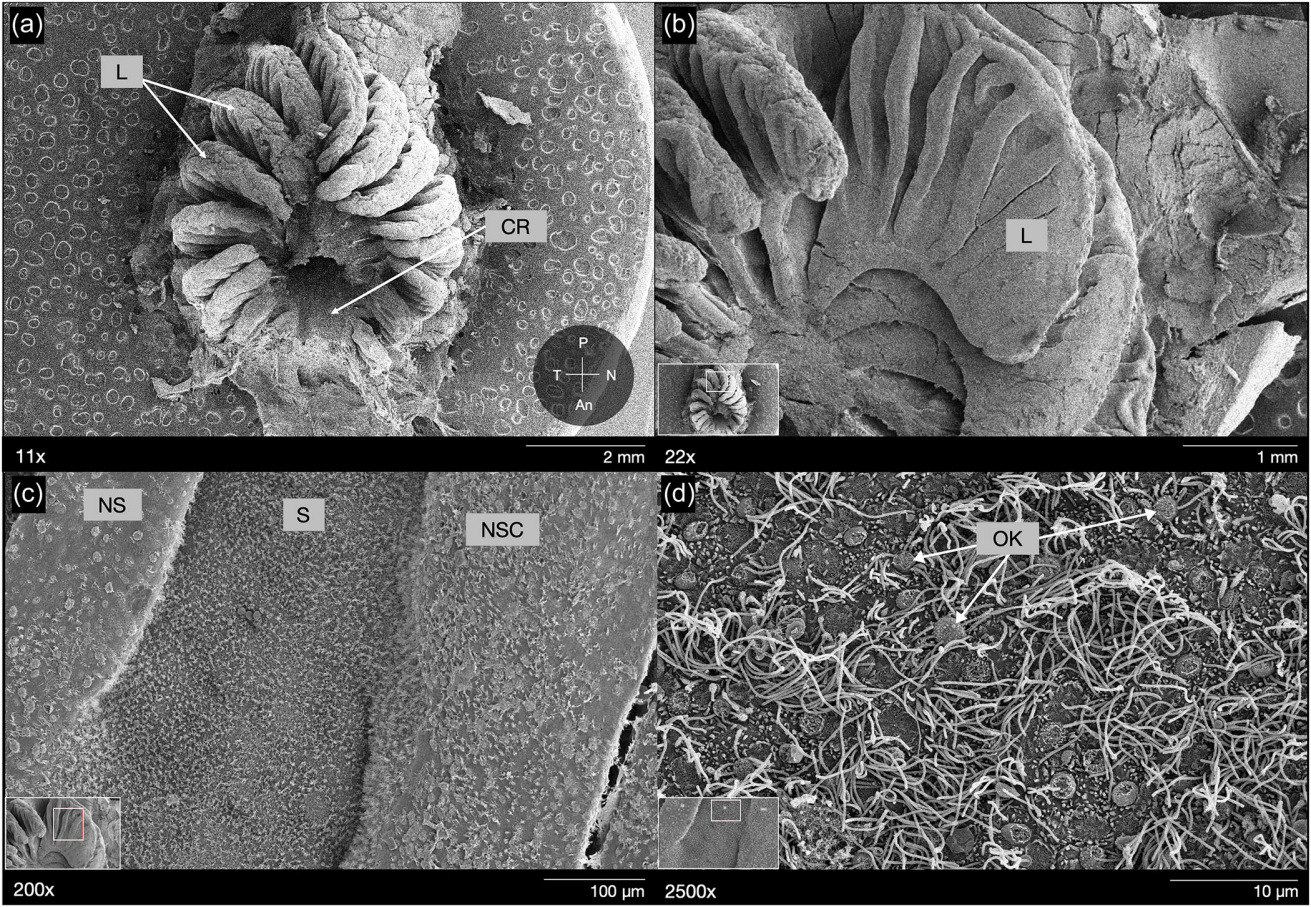
*Oncorhynchus nerka*, scanning electron micrographs of the olfactory rosette of a representative adult specimen. (a) Entire rosette at ×11 magnification, indicating the lamellae (L) and central raphe (CR) (b) Adult lamellae (L), showing the presence of secondary folding along an individual lamella at ×22 magnification. (c) Sensory epithelia of a single lamellae, where secondary folding is present at ×200. Sensory (S) and nonsensory (NS) regions indicated, as well as regions containing nonsensory, “kinociliated” cells (NSC). (D) Sensory epithelia appear to contain ciliated and microvillous ORN types. Olfactory knobs (OKs) are dispersed throughout the sensory epithelia at ×2500. Orienting images in the lower left corner indicate where an increase in magnification took place along the rosette for each subsequent panel. Orientations shown in (a) indicates anterior (An), nasal (N), posterior (P), and temporal (T) position of the left rosette within the nasal cavity across all panels. ORN, olfactory receptor neuron.

**Figure 6:**
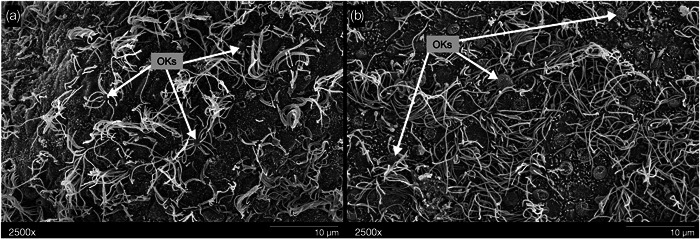
*Oncorhynchus nerka*, scanning electron micrographs, presence of olfactory knobs (OKs) on the sensory epithelia of a representative (a) fry and (b) adult specimen. OKs are indicated by white arrows.

Two types of epithelia were found on the rosette of fry and adult specimens: a nonsensory epithelium, populated with large supporting cells that bear microvilli or cilia (“kinociliated cells”; e.g., Cox, 2008), and a sensory epithelium, populated by ciliated and microvilli‐bearing supporting cells that surrounded the apical OKs of ORNs (Figure 5b–d). However, the distribution of the two epithelial regions and the organization and composition of the sensory region differed between fry and adult specimens. In fry, nonsensory regions were found only along the peripheral edges of the rosette itself, and the visible portions of the lamellae were fully covered with a sensory epithelium that appeared evenly distributed, with no secondary lamellae (Figure 2a). In adults, the secondary lamellae were prominent; the sensory epithelium was distributed within the troughs of the secondary lamellae, while the nonsensory regions extended from the inner lamellar margins to the outer lamellar margins and even to the secondary lamellar edges (Figure 5b,c). For both life stages, the sensory epithelium appeared populated with the two types of supporting cells (ciliated and microvillous), intertwined with the presence of OKs. However, in fry, the sensory epithelium was densely packed with OKs of a similar appearance that did not appear homogeneously distributed across the lamellar surface, while the remaining sensory area was covered by cilia‐bearing supporting cells (×2500; Figure 6a). The adult sensory epithelium comprised more dispersed OKs, which qualitatively appeared more evenly distributed than in fry. Although they could not be empirically identified, apparent variation in OKs was suggestive of multiple cell types across the epithelial surface (Figure 6b).

## 4 DISCUSSION

The peripheral olfactory system of fishes is well‐developed and highly variable in shape and organization across species (Cox, 2008; Hara, 2011; Kasumyan, 2004). Considerable interspecific variation has been documented in the gross morphology, anatomy, and ultrastructure of the fish peripheral olfactory system. These include differences in the presence and size of the nasal bridge between nostrils, total rosette size, lamellar arrangement within the rosette, lamellae number and lamellar surface area, and the total number, density, distribution, and type of ORNs across the sensory epithelium (Døving et al., 1985; Hansen & Zielinski, 2005; Kasumyan, 2004; Yamamoto, 1982; Zeiske et al., 1992), as well as variation in the size and shape of the olfactory bulbs themselves (Kotrschal et al., 1998; Wagner, 2001; Yopak et al., 2015, 2019) within and across fish groups. However, while interspecific variation in olfactory anatomy has been reasonably well explored in fishes, intraspecific diversity in this system is less understood (e.g., Bauchot et al., 1979; Brandstätter & Kotrschal, 1989; Kihslinger et al., 2006; Näslund et al., 2017; Tomoda & Uematsu, 1996).

Consistent with our predictions, morphological changes occur in the peripheral olfactory organ of sockeye salmon throughout ontogeny, which may underlie imprinting, migration, and/or natal homing in this species. In accordance with the assumption that larger individuals have larger olfactory organs (Hansen & Zielinski, 2005), both RS and complexity (lamellar number and morphology, epithelial distribution, and composition) change throughout life in this species. The olfactory rosette scaled significantly with body size, whereby overall RS continues to increase as body size increases (Figure 4). Correspondingly, there was a steady increase in lamellae number across life stages (Figure 3) Similarly, in Arctic char, *Salvelinus alpinus*, lamellar number increased up to a maximum of 10–15 lamellae per rosette, 30 months posthatching (Olsen, 1993). Development of new lamellae during early life history stages likely initiates from the rostro‐basal portion of the olfactory rosette, where the smallest lamellae are found (Kasumyan, 2004; Theiss et al., 2009). Lamellae number is believed to be species‐specific in some fish groups (e.g., Ferrando et al., 2019), but our results suggest some consistency in ontogenetic growth of the rosette across some members of the *Oncorhynchus* genus, including chum and masu salmon, *O. masou*, whereby lamellae number also increases throughout ontogeny (Kalinina et al., 2005; Kudo et al., 2009; Yamamoto & Ueda, 1977).

The relationship between lamellar growth in the peripheral rosette, olfactory processing in the brain, and memory formation is poorly understood. Evidence suggests that salmon imprint to chemosensory patterns during juvenile stages (Dittman et al., 1996; Dittman et al., 2015; Yamamoto et al., 2013) that can then be utilized later in life to identify their natal stream as migrating adults. Imprinting occurs at the parr‐smolt transformation in several salmonids (e.g., Hasler & Scholtz, 1983, Dittman et al., 1996), but evidence also indicates that some aspects of imprinting may occur as early as the embryonic and hatching stages (53–63 days postfertilization: Dittman et al., 2015; Havey et al., 2017; Tilson et al., 1994). Therefore, the presence of specific lamellae as early as the fry stage may be critical for imprinting. In this case, as mature adult sockeye complete their homing migrations, only ~7 of the 14–15 lamellae within the rosette would have been present at the time of imprinting, similarly proposed for chum salmon (Kudo et al., 2009). However, there is currently no empirical evidence that links specific lamellae to imprinting mechanisms, so this requires further study. In addition to changes in lamellae number throughout ontogeny, lamellar size also varies in sockeye salmon individuals. For all life stages, a qualitative assessment of visible portions of the rosette suggests the posterior lamellae are the most well‐developed (i.e., the largest), as compared to those positioned more anterior in the nare (Figure 2), which is consistent with olfactory rosette anatomy in other teleost fishes (Chakrabarti & Ghosh, 2011; Dymek et al., 2021; Ferrando et al., 2019; Hansen & Zeiske, 1998; Hansen & Zielinski, 2005; Kudo et al., 2009).

**Figure 7:**
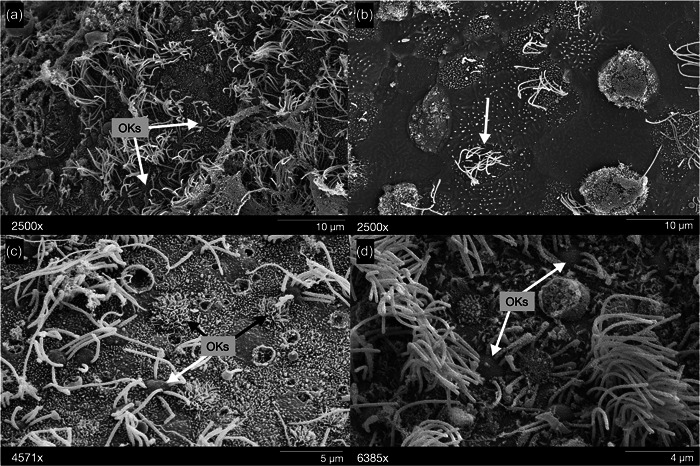
*Oncorhynchus nerka*, scanning electron micrographs of the olfactory epithelia of representative specimens of (a and b) fry and (c and d) adult sockeye salmon, showing the diversity in olfactory knobs (OKs) of olfactory receptor neurons (ORNs) and supporting cells along the sensory epithelium (b). (a) A small, rounded knob bearing cilia (white arrowhead). (b) Arrow indicates supporting cells. (c) OKs covered in microvilli (black arrowhead) and small, rounded, knob‐bearing cilia (white arrowhead). (d) Small, rounded, knob‐bearing cilia (white arrowhead). Scale bars: (a) 10 µm, (b) 10 µm, (c) 5 µm, (d) 4 µm.

Distinct apical OKs of the ORNs were identified within the sensory epithelium in sockeye salmon (Figure 7). Variation in the density and distribution of these OKs along the epithelial surface may provide insight into changes in olfactory capacity at these distinct life history stages (Ahuja et al., 2014; Oka et al., 2012; Shoji et al., 2000). There are five main ORN morphotypes currently described in teleosts: ciliated, microvillous, crypt (Fishelson et al., 2010; Kermen et al., 2013; Muller, 1984), kappe (Ahuja et al., 2014), and pear (Calvo‐Ochoa & Byrd‐Jacobs, 2019). Although empirical identification of individual subtypes was outside of the scope of this study, as they cannot be confirmed without immunohistochemistry (e.g., Camilieri‐Asch, Yopak, et al., 2020), the presence of OKs with apparent morphological differences between fry and adult specimens suggests there may be ontogenetic changes in ORN subtype number and density. However, it is important to approach this with caution, as morphological differences from SEM can be due to fixation artifacts or tissue preparation, rather than true anatomical variation. In fry, the sensory epithelium was densely packed with OKs of a similar appearance that did not appear to be homogeneously distributed across the lamellar surface, while the remaining sensory area was covered by cilia‐bearing supporting cells (×2500; Figure 6a). Additional OKs may have been present, but potentially obscured by the dense cilia of the supporting cells (Figures 6 and 7). In contrast, the sensory epithelium in adults presented a more dispersed and morphologically diverse OK distribution, which qualitatively appeared more evenly distributed than in fry (Figure 6b). Critically, future work should empirically identify and quantify ORN subtype number and density throughout ontogeny, which may inform the relative capacity to bind different odorant classes (e.g., amino acids, bile salts, pheromones) at these key life stages.

Not all teleost fishes exhibit secondary lamellar folds (currently only described in Salmoniformes, Gadiformes, Esociformes, many species of Anabantiformes, and some species of Perciformes (Kasumyan, 2004). In sockeye, during the fry, parr, and smolt life stages, no secondary lamellae were observed (Figure 2a–c), but they were evident in the adults (Figures 2d and 5). This is similar to chum salmon (Kudo et al., 2009), in which there is a distinct shift in lamellar complexity, with the appearance of secondary lamellae in mature adults. The lack of secondary lamellae in juvenile sockeye salmon suggests a lesser total lamellar surface area compared to adults, which may reflect differences in ORN number (Kudo et al., 2009). In chum salmon, young individuals have fewer ORNs (180,000) compared to mature individuals (14.2 million; Kudo et al., 2009), though overall ORN density may not change (Kalinina et al., 2005). However, while secondary lamellae may increase total lamellar surface area, ORN distribution is not homogenous and sensory epithelium was not identified on the peaks of the secondary folds in adult sockeye. Similarly, previous work in salmonids showed that the convex areas of the secondary folds lack ORNs (Kudo et al., 2009; Olsen, 1993; Yamamoto & Ueda, 1977). This is in contrast to many elasmobranch fishes, where the sensory epithelium covers the secondary lamellae (both troughs and peaks), with areas of nonsensory epithelium along the inner margins of the lamellae and/or as intermittent projections into the sensory regions in some species (e.g., Camilieri‐Asch, Shaw, et al., 2020; Schluessel et al., 2008; Theiss et al., 2009; Simonitis & Marshall, 2022). In adult sockeye salmon, as in other salmonids, the sensory epithelium lies only along the troughs of the secondary folds, while the nonsensory epithelium covers the secondary lamellar peaks (Figure 5c). Thus, secondary lamellae in adults may not serve to expand olfactory capacity, but rather may facilitate water dynamics in the olfactory capsule for efficient odor sampling (Kudo et al., 2009). Although fry have fewer lamellae, lack secondary folds, and likely possess a smaller absolute lamellar surface area, the high relative proportion of sensory epithelium and scattered distribution of ORNs throughout the epithelial surface might enable them to maximize the existing surface area for chemosensory sampling across multiple ORN subtypes during imprinting.

## 5 CONCLUSIONS

The peripheral olfactory system in sockeye salmon undergoes several morphological changes across ontogeny. The increase in size and complexity of the olfactory rosette, appearance of secondary folds, and apparent difference in ORN distribution across the epithelial surface suggests a potential shift in olfactory capacity between life stages, providing support for the hypothesis that sockeye salmon rely on chemosensory cues to facilitate natal migration as adults. Future research should include quantification of ORN number, subtype abundance, proliferation rate, and distribution across the rosette, coupled with simulations of fluid dynamics within the olfactory cavity, across distinct life stages. This would allow us to more finely resolve the changes in the olfactory system in sockeye salmon, and ultimately help us to better understand the critical odorants guiding them home.

## AUTHOR CONTRIBUTIONS

**Sarah E. Rheinsmith**: Formal analysis; data curation; methodology; conceptualization; writing ‐ original draft; investigation; visualization; writing – review & editing. **Thomas P. Quinn**: Data curation; writing – review & editing; validation. **Andrew H. Dittman**: Writing – review & editing; validation; data curation. **Kara E. Yopak**: Supervision; writing – review & editing; project administration; methodology; conceptualization; funding acquisition; data curation.

## Data Availability

The data that support the findings of this study are available on request from the corresponding author. The data are not publicly available due to privacy or ethical restrictions.
